# Prevalence and predicting factors for commonly neglected sexual side effects to brachytherapy for prostate cancer: a cross-sectional observational study

**DOI:** 10.1093/sexmed/qfad064

**Published:** 2023-12-06

**Authors:** Sami Beji, Alexander Bjørneboe Nolsøe, Christian Fuglesang S Jensen, Peter Busch Østergren, Jens Sønksen, Rasmus Bisbjerg, Henrik Jakobsen, Mikkel Fode

**Affiliations:** Department of Urology, Copenhagen University Hospital, Herlev and Gentofte Hospital, Herlev, 2730, Denmark; Department of Urology, Copenhagen University Hospital, Herlev and Gentofte Hospital, Herlev, 2730, Denmark; Department of Clinical Medicine, University of Copenhagen, Copenhagen, Denmark; Department of Urology, Copenhagen University Hospital, Herlev and Gentofte Hospital, Herlev, 2730, Denmark; Department of Urology, Copenhagen University Hospital, Herlev and Gentofte Hospital, Herlev, 2730, Denmark; Department of Clinical Medicine, University of Copenhagen, Copenhagen, Denmark; Department of Urology, Copenhagen University Hospital, Herlev and Gentofte Hospital, Herlev, 2730, Denmark; Department of Clinical Medicine, University of Copenhagen, Copenhagen, Denmark; Department of Urology, Copenhagen University Hospital, Herlev and Gentofte Hospital, Herlev, 2730, Denmark; Department of Urology, Copenhagen University Hospital, Herlev and Gentofte Hospital, Herlev, 2730, Denmark; Department of Urology, Copenhagen University Hospital, Herlev and Gentofte Hospital, Herlev, 2730, Denmark; Department of Clinical Medicine, University of Copenhagen, Copenhagen, Denmark

**Keywords:** climacturia, brachytherapy, orgasm, penile shortening, prostate cancer, sexual dysfunction

## Abstract

**Background:**

Low-dose-rate brachytherapy (LDR-B) is an established treatment for localized prostate cancer. However, while erectile function is relatively well documented, other changes in sexual function are sparsely investigated.

**Aim:**

The study sought to investigate orgasmic dysfunction, urinary incontinence during sexual activity (UIS), changes in penile morphology, and sensory disturbances in the penis following LDR-B.

**Methods:**

A cross-sectional questionnaire-based study in patients who underwent LDR-B at our center from 2010 to 2020. The questionnaire included the International Index of Erectile Function–Erectile Function Domain (IIEF-EF) and questions on orgasm, UIS, changes in penile morphology, and penile sensory disturbances.

**Outcomes:**

Outcomes were prevalence rates of altered perception of orgasm, orgasm associated pain, anejaculation, UIS, alterations in penile morphology, penile sensory disturbances, and predictors of these side effects.

**Results:**

Overall, 178 patients responded to the questionnaire. The median age was 70 years (range, 51-83 years), and the median time since LDR-B was 93 months (range, 21-141 months).

Overall, 142 (80%) were sexually active and 126 (70.8%) had erectile dysfunction (ED). Of the sexually active patients, 8 (5.6%) reported anejaculation and 7 (4.9%) reported anorgasmia. Another 67 (46.9%) had decreased orgasmic intensity, while 69 (49.3%) reported an increased time to orgasm. Twenty-six (18.3%) patients had experienced orgasm-associated pain with a median visual analog pain score of 2. Considering overlap, 44 (31.0%) patients had an unchanged orgasmic function. Six (3.3%) patients had experienced UIS at least a few times. Penile length loss was reported by 45 (25.2%) patients. Seventeen (9.6%) patients reported an altered curvature of their penis and 9 (5%) had experience painful erection. Thirty-three (18.5%) patients had experienced decreased penile sensitivity. On multivariate analyses, ED was the only independent risk factor for altered perception of orgasm (odds ratio [OR], 6.6; *P* < .0001), orgasmic pain (OR, 5.5; *P* = .008), and penile shortening (OR, 4.2; *P* < .0056). No independent risk factors were identified for UIS or sensory penile disturbances.

**Clinical implications:**

Patients undergoing LDR-B should be adequately informed about possible side effects, and clinicians should inquire about these during follow-up visits.

**Strength and Limitations:**

We are the first to comprehensively explore the previously neglected side effects of LDR-B for prostate cancer. Limitations are the cross-sectional design assessing the cohort at different time points following their treatment and the response rate.

**Conclusions:**

Orgasmic dysfunction, changes in penile morphology, and sensory disturbances in the penis are common side effects of LDR-B for prostate cancer. UIS is only experienced by a small minority.

## Introduction

Prostate cancer is the second most frequently diagnosed cancer in men worldwide.^1^ Curative treatment for localized prostate cancer includes radical prostatectomy (RP) and external beam radiation therapy (EBRT).^2^ The cancer-specific survival is excellent with both modalities; however, they are associated with several side effects including urinary problems, erectile dysfunction (ED), orgasmic and ejaculatory disturbances, and changes in penile morphology and sensitivity.^3^ Another option of curative treatment is internal radiation with low-dose-rate brachytherapy (LDR-B). This treatment is primarily offered to men with intermediate risk localized prostate cancer and uses implanted radioactive sources in close contact to the tumor to deliver a high dose of radiation to the affected tissue (144 Gy) without passing through surrounding normal tissue as in EBRT. Therefore, LDR-B has relatively minimal short- and long-term morbidity with high rates of erectile preservation and low rates of genitourinary toxicity.^4^ However, while erectile function is relatively well documented, other changes in sexual function are sparsely investigated. We decided to conduct this study to comprehensively evaluate sexual side effects of LDR-B. This is important to inform future patients and help them decide between different treatment modalities for prostate cancer. Further, it may serve to make clinicians aware of various side effects, which should be addressed following treatment.

We sought to investigate orgasmic and ejaculatory dysfunction, orgasm-associated pain (OAP), urinary incontinence during sexual activity (UIS), changes in penile morphology, and sensory disturbances in the penis in patients with prostate cancer treated with LDR-B. We hypothesized that these side effects do occur after LDR-B but at a lower rate compared with those after RP and EBRT.

## Methods

This study was a part of a single-center, cross-sectional, questionnaire-based investigation. Men who underwent LDR-B at Herlev Hospital from March 2010 to February 2020 were eligible for inclusion. Respondents were excluded from the analyses if they had received subsequent treatments of their prostate cancer in the form of RP, EBRT, and/or castration therapy. All patients were treated with low-dose iodine-125 permanent implantation (loose) seeds (BrachySource; Bard Medical) with a prescribed minimum peripheral dose of 144 Gy as previously described.^5^ Briefly, real-time planning was used, and seeds were implanted guided by ultrasound with a peripheral loading technique.

We used an adapted version of a previously developed questionnaire on sexual side effects following prostate cancer treatment.^6^ This included questions on demographics, comorbidities, and pretreatment sexual function. For assessment of sexual side effects, the questionnaire included the International Index of Erectile Function–Erectile Function domain (IIEF-EF)^7^ and 15 purpose-built questions on perception of orgasm, OAP, UIS, changes in penile morphology, and sensory disturbances in the penis. Patients were able to give either yes/no answers or respond via a 5-point Likert scale. Visual analog scales from 1 to 10 were used to report bother and pain. Changes in penile length were assessed subjectively by patients. These questions are not formally validated. Their validity and reproducibility have previously been tested by interviews with patients and a test–retest procedure.^6^ The International Consultation on Incontinence Questionnaire Short Form (ICIQ-SF) (https://iciq.net/europe)^8^ was used to assess urinary function. Regarding UIS, patients were asked to differentiate between a loss of urine during climax (climacturia) or during other parts of sexual activity. Eligible patients were identified from hospital records and contacted through secure mail in November 2021. Two months later, nonresponders received a reminder with an identical questionnaire. Information on disease characteristics and the duration and time of LDR-B was extracted from medical records of the participants.

Descriptive statistics were used to summarize patient and disease characteristics as well as reported sexual side effects. To assess possible predictors of the various side effects, univariate logistic regression analyses were performed, and if multiple covariates were found predictive for a given side-effect, they were combined in a multivariate logistic regression analysis. For statistical strength in the logistic regression analyses, altered perception of orgasm was defined as increased time to climax, decreased orgasm intensity, anorgasmia, or any combination of the 3. UIS was defined as urine lost at least a few times during any part of sexual activity. All penile sensory disturbances were pooled, and penile shortening was considered as a binary occurrence (yes or no). Covariates included in the analyses were predefined (Table 1), and outcomes of logistic regression analyses were calculated as odds ratios (ORs), 95% confidence intervals (CIs), and *P* values. Two-sided *P* values <.05 were considered statistically significant. All statistical analyses were performed with SAS statistical software 9.4M7 (SAS Institute).

**Table 1:** Demographics and IIEF-EF and ICIQ-SF scores at the time of the survey and D’Amico score before treatment.

Age, y	70 (51-83)
Time since LDR-B, mo	93 (21-141)
Charlson Comorbidity Index	0 (0-5)
BMI, kg/m^2^	27.2 (20.4-40.9)
IIEF-EF	20 (1-30)
ICIQ-SF	0 (0-20)
D’Amico risk classification	
Low	40
Intermediate	137
High	1

Values are mean (range) or n.

Abbreviations: BMI, body mass index; ICIQ-SF, International Consultation on Incontinence Questionnaire Short Form; IIEF-EF, International Index of Erectile Function–Erectile Function Domain LDR-B, low-dose-rate brachytherapy

The study was approved by the Danish Data Protection Agency (ID P-2020-710). Informed consent was obtained as part of the questionnaire. The manuscript was prepared according to the STROBE (Strengthening the Reporting of Observational Studies in Epidemiology) statement.

### Main outcome measures

The main outcome measures were the prevalence rates of altered perception of orgasm, OAP, anejaculation, UIS, alterations in penile morphology, and sensory disturbances in the penis. Secondary outcome measures were predictors of these side effects as identified by multivariate logistic regression analyses.

## Results

A total of 389 patients who had received LDR-B with curative intent in the predetermined period of time were invited to participate. A total of 184 (47%) agreed to participate in the survey and responded to the questionnaire. Two men had received subsequent EBRT, 1 had undergone RP, and 3 had received medical castration therapy. These patients were excluded from further analyses and 178 men were included.

The median age of the included patients was 70 years (range, 51-83 years), and the median time since LDR-B was 93 months (range, 21-141 months). Further demographics are presented in Table 1 along with IIEF-EF and ICIQ-SF scores at the time of the survey. Regarding pretreatment function, 36 (20%) reported to have had ED before LDR-B, 8 (4%) reported a lack of ejaculation and orgasm, 8 (4%) had pain with their orgasms, 2 (1%) had experienced incontinence in relation to sex, and 2 (1%) reported to have had a curved penis, while no one reported to have had painful erections.

Overall, 126 (71%) participants reported ED at the time of the survey with 49 (28%) experiencing mild ED, 20 (11%) moderate ED, and 57 (32%) severe ED according to the IIEF-EF. Of the 178 patients, 142 (80%) had been sexually active in the previous 3 months.

Of the sexually active patients, 8 (6%) reported anejaculation, and 7 (5%) patients reported anorgasmia. Another 67 (47%) reported a decreased intensity of their orgasms, while 69 (49%) reported that the time it took to reach orgasm had increased. No patients reported an increased intensity of their orgasms, but 8 (6%) reported that the time to orgasm had decreased. Considering overlap in the mentioned groups, 44 (31%) patients reported that their orgasmic function was unchanged. Twenty-six (18%) patients had experienced OAP at least a few times, with a median visual analog pain score of 2 (range, 1-8). Fourteen patients reported that the pain lasted for up to 1 minute, 2 patients had pain lasting between 1 and 10 minutes, and 3 patients reported that the pain lasted for more than 10 minutes.

Six (4%) patients had experienced UIS at least a few times after LDR-B with a small or moderate amount of urine lost. Two patients had experienced the loss of urine only during foreplay/intercourse, 2 patients experienced the loss of urine only during orgasm, and the remaining 2 reported a loss of urine both during intercourse and in conjunction with orgasm.

In the whole group (N = 178), a subjective penile length loss was reported by 45 (25%) patients. Seven patients reported loss under 1 cm, 32 patients reported a 1- to 3-cm loss, 5 patients reported a 3- to 5-cm loss, and 1 did not report the lost length. Seventeen (10%) patients reported an altered curvature of their penis, and 9 (5%) patients reported that they had experience painful erections with a median visual analog pain score of 4 (range, 2-7). Only 3 patients reported both an altered curvature and pain. Thirty-three (19%) patients had experienced decreased sensitivity in the penis after LDR-B, 2 (1%) reported a cold sensation, 2 (1%) reported a warm sensation, and 9 (5%) reported paresthesia. Three (2%) patients had experienced an increased sensitivity. A summary of the neglected sexual side effects is presented in Table 2.

**Table 2:** Summary of neglected sexual side effects.

Sexually active responders (n = 142)	
Anejaculation	8 (6)
Anorgasmia	7 (5)
Decreased intensity of orgasm	67 (47)
Increased time to orgasm	69 (49)
Decreased time to orgasm	8 (6)
OAP	26 (18)
UIS	6 (4)
Responds from entire cohort (n = 178)	
Erectile function	
No ED	52 (29)
Mild ED	49 (28)
Moderate ED	20 (11)
Severe ED	57 (32)
Subjective penile length loss	
<1 cm	7 (4)
1-3 cm	32 (18)
3-5 cm	5 (3)
>5 cm	0
Altered curvature of the penis	17 (10)
Painful erection	9 (5)
Altered sensitivity of the penis	
Decreased sensitivity	33 (19)
Cold sensation	2 (1)
Warm sensation	2 (1)
Paresthesia	9 (5)
Increased sensitivity	3 (2)

Values are n (%).

Abbreviations: ED, erectile dysfunction; OAP, orgasm-associated pain; UIS, urinary incontinence during sexual activity.

On univariate analyses, ED and ICIQ-SF score affected the risk of an altered perception of orgasm. However, on multivariate analyses, only ED remained an independent risk factor, with an OR of 6.6 (95% CI, 2.9-15.0; *P* < .001). Likewise, the presence of ED increased the risk of OAP, with an OR of 5.5 (95% CI, 1.6-19.3; *P* = .008). On univariate analyses, ED and body mass index affected the risk of penile shortening, but again only ED remained an independent risk factor for penile shortening on multivariate analysis with an OR of 4.2 (95% CI, 1.5-11.2; *P* = .006). No independent risk factors were identified for UIS or sensory penile disturbances. There was no apparent influence of pretreatment sexual problems, as these were persistent in some men and had disappeared in others.

## Discussion

Internal radiation with low-dose brachytherapy has shown excellent overall and disease-specific survival in low- and intermediate-risk localized prostate cancer, presumably with fewer sexual side effects.^5^ This is the first study to perform a comprehensive investigation of these side effects. Our results show that while a large proportion of the patients do experience changes in their orgasmic function, this is usually limited to decreased intensity and/or an increase in the time it takes to reach orgasm. The prevalence of anejaculation and anorgasmia was almost identical before and after LDR-B. Importantly, orgasmic pain had been experienced by <1 in 5 patients and was usually mild and of short duration. Both climacturia and UIS in general were rare. Likewise, subjective penile morphological changes were limited, except for subjectively reported minor length loss in a quarter of the patients, while decreased penile sensitivity was a common sensory disturbance. Very few patients experienced a combination of penile curvature and pain indicative of Peyronie’s disease.

Few studies have investigated sexual side effects other than ED in men undergoing LDR-B. A study published by Huyghe et al^9^ in 2009 concentrated on orgasmic function in 241 sexually active men. Here, posttreatment anejaculation was seen in 18.7% and the ejaculatory volume was diminished in the majority of the remaining patients. Ten percent experienced anorgasmia and 30.3% of the patients experienced painful ejaculations. When compared with our study, there is a striking difference in the number of patients who experience pain. This may in part be accounted for by a relatively high pretreatment occurrence of painful ejaculation (12.9% of patients) in the Huyghe et al study. Further, the occurrence was associated with an unspecified greater number of implanted needles, indicating that the radiation dose and delivery may play a key role. The latter could also play a role in the high occurrence of anejaculation in the Huyghe et al study. Another study from 2017 by Gay et al^10^ reported that 43.9% had poor, very poor, or no ability to achieve orgasm 2 years after brachytherapy compared with 23.3% at baseline. This corresponds well with 49% of our patients experiencing delayed orgasm and 47% experiencing decreased orgasmic sensation. Meanwhile, when counting all men who experienced altered orgasmic function in our study, 69% were affected by some sort of orgasmic change.

When comparing brachytherapy with other treatments for prostate cancer, these sexual side effects seem to be less frequent. Thus, a previous study using the same methods and research questions in 109 sexually active men who had undergone EBRT found that 24% percent reported anorgasmia, 11% reported anejaculation, and 15% reported OAP.^11^ Meanwhile, UIS, subjective penile length loss, and penile sensory disturbances were comparable to what we have seen after brachytherapy. It is interesting that sensory disturbances in the penis increased with time in that study, suggesting that EBRT has late toxic effects on sexuality, which we did not observe in our LDR-B cohort.

Another very similar trial in men following RP (N = 256) found that, despite universal anejaculation, the rate of anorgasmia was only 5%.^6^ Meanwhile, a feeling of decreased orgasmic intensity and/or increased time to reach orgasm was experienced by the vast majority of patients, and 10% reported pain during orgasm. Importantly, 38% were bothered by UIS, which was uncommon after brachytherapy, and almost half of the participants reported a subjective loss of penile length. As seen in our study, ED increased the risk of the latter.

Only a few other studies have dealt with the so-called neglected sexual side effects to EBRT. Although these are more difficult to compare due to variations in methodology, the findings are generally similar, with approximately half the sexually active patients experiencing a decreased sensation of orgasm, 16% experiencing anejaculation, and 10% feeling pain with ejaculation.^12-14^ One study (N = 110) also found a 5.2% incidence of UIS following EBRT.^15^ As EBRT is often combined with androgen deprivation therapy, this may also play a role and should be kept in mind when comparing with brachy therapy. Regarding RP, we have more studies available, but the rates of side effects vary widely. Thus, anorgasmia is seen in 20% to 37%,^16^^,^^17^ decreased orgasmic intensity in 20% to 65%,^16^^,^^18-20^ OAP in 3% to 19%,^16^^,^^20-24^ UIS in 20% to 93%,^21^^,^^22^^,^^25^ and penile shortening in 0% to 55%.^23^^,^^26-28^

The pathogenesis behind orgasmic disturbances, UIS, and changes in penile morphology and sensitivity are all unclear and have been a subject of debate.^29^ A reported correlation between orgasmic function and erectile function suggests that anorgasmia and reduced orgasmic intensity after prostate cancer treatment may be secondary to reduced tactile stimulation of the dorsal penile nerve, as its fibers are less exposed with reduced engorgement of the glans penis. This is corroborated by the finding that orgasmic function improves with ED treatment following RP.^30^ It also poses a possible explanation as to why some patients experience reduced penile sensitivity. Meanwhile, orgasmic pain has been suggested to stem from increased muscle tone in the pelvic floor muscles and/or spastic contractions of the semen pathways upon orgasm.^28^ Theoretically, this may arise following both surgery and radiation of the area but seems to be especially pronounced after surgery with seminal vesicle sparing.^28^

It has been reported that UIS after RP is associated with decreased functional urethral length.^31^ Further, surgery may cause direct damage to the urethral sphincter and to its nerve fibers, which may explain both the great variation in incidence after RP and the rarity of the problem following EBRT and LDR-B. Finally, penile shortening has been linked to ED in several studies and is believed to be caused by resulting hypoxia with subsequent smooth muscle loss and fibrosis.^32^^,^^33^ In line with these theories, ED was the only independent risk factor for any of the previously mentioned side effects in our study, as it predicted an altered perception of orgasm, orgasmic pain, and subjective penile shortening. This supports the notion that orgasmic changes may simply be a result of reduced sensory stimulation during sex, while shortening may be caused by progressive penile fibrosis due to a lack of tissue oxygenation. This explanation would account for the occurrence of similar sexual side effects in very different treatment modalities for prostate cancer. The apparent link between ED and orgasmic pain is difficult to explain but may simply be an indicator of a relatively high degree of local tissue affection in some patients resulting in both problems.

At our center, erectile function is evaluated before and after curative prostate cancer treatments including brachytherapy. ED treatments are offered following treatment and prescribed on an individual basis. Generally, the first choice is phosphodiesterase type 5 inhibitors with injection therapy and/or vacuum devices offered in case of treatment failure. A penile rehabilitation program is not offered, as high-quality studies have failed to show an impact on long-term erectile function following RP and EBRT.^34-36^ To our knowledge, no randomized trials have explored the concept in conjunction with brachytherapy.

The main strength of our study is that we are the first to comprehensively explore the so-called neglected side effects in LDR-B for prostate cancer. Further, we have used a similar methodology as in previous studies on RP and EBRT, which have included a validation process of the questionnaire. This makes comparisons across trials relatively simple. However, our study also contains limitations. The most obvious is the cross-sectional design assessing the cohort at different time points following their treatment. The retrospective assessment of sexual bother before brachytherapy and the long follow-up time mean that in some cases the problems could have arisen due to other factors. Further, the response rate of 47% introduces a risk of attrition bias. In this regard, the proportion of sexually active men in our survey makes it likely that the group is overrepresented. In theory, it could cause an underestimation of the most bothersome side effects, such as UIS, as some men may have seized sexual activity due to these. However, this is an inherent limitation to cross-sectional studies investigating sexual side effects, which has also been seen in previously published studies. Regarding other parameters such as tumor characteristics and pretreatment sexual and urinary function, there is unlikely to be significant differences between responders and nonresponders due to our department’s criteria for offering brachytherapy. Regarding the external validity of the study, our results are likely representable for LDR-B performed with a similar protocol in other centers. However, the results should not be generalized to centers who perform the treatment in a different manner, and the incidence of the reported side effects could be noticeably higher following high-dose-rate brachytherapy. Further limitations include the lack of formal validation of the study questionnaire and the large span in follow-up time for the included patients.

## Conclusion

Orgasmic dysfunction, changes in penile morphology, and sensory disturbances in the penis are common side effects of LDR-B for prostate cancer but are usually mild in nature. Meanwhile, UIS is only experienced by a small minority of patients. Patients should be informed of the risk of these side effects prior to treatment and clinicians should routinely address them during follow-up.
